# Biomass Production of the EDEN ISS Space Greenhouse in Antarctica During the 2018 Experiment Phase

**DOI:** 10.3389/fpls.2020.00656

**Published:** 2020-05-26

**Authors:** Paul Zabel, Conrad Zeidler, Vincent Vrakking, Markus Dorn, Daniel Schubert

**Affiliations:** EDEN Research Group, German Aerospace Center (DLR), Institute of Space Systems, Bremen, Germany

**Keywords:** bio-regenerative life support system (BLSS), plant cultivation chamber, space plant growth facility, space food and nutrition, space analog studies, controlled environment agriculture (CEA)

## Abstract

The EDEN ISS greenhouse is a space-analog test facility near the German Neumayer III station in Antarctica. The facility is part of the project of the same name and was designed and built starting from March 2015 and eventually deployed in Antarctica in January 2018. The nominal operation of the greenhouse started on February 7th and continued until the 20th of November. The purpose of the facility is to enable multidisciplinary research on topics related to future plant cultivation on human space exploration missions. Research on food quality and safety, plant health monitoring, microbiology, system validation, human factors and horticultural sciences was conducted. Part of the latter is the determination of the biomass production of the different crops. The data on this topic is presented in this paper. During the first season 26 different crops were grown on the 12.5 m^2^ cultivation area of the greenhouse. A large number of crops were grown continuously throughout the 9 months of operation, but there were also crops that were only grown a few times for test purposes. The focus of this season was on growing lettuce, leafy greens and fresh vegetables. In total more than 268 kg of edible biomass was produced by the EDEN ISS greenhouse facility in 2018. Most of the harvest was cucumbers (67 kg), lettuces (56 kg), leafy greens (49 kg), and tomatoes (50 kg) complemented with smaller amounts of herbs (12 kg), radish (8 kg), and kohlrabi (19 kg). The environmental set points for the crops were 330–600 μmol/(m^2*^s) LED light, 21°C, ∼65% relative humidity, 1000 ppm and the photoperiod was 17 h per day. The overall yearly productivity of the EDEN ISS greenhouse in 2018 was 27.4 kg/m^2^, which is equal to 0.075 kg/(m^2*^d). This paper shows in detail the data on edible and inedible biomass production of each crop grown in the EDEN ISS greenhouse in Antarctica during the 2018 season.

## Introduction

Food production during human space missions to and on Moon and Mars is a necessary step to reduce resupply mass from Earth and thus long-term mission costs. Growing plants for food production also offers the advantages of producing oxygen and removing carbon dioxide from the atmosphere as well as the recycling of water. Because of these advantages, experiments in growing plants in space began as early as the first manned space stations and continue to the present day (Zabel et al., 2016a).

Several research teams conducted experiments in cultivating plants in a closed controlled environment on Earth for the application in future space missions in the past (Wheeler, 2017). Notable are NASA’s Biomass Production Chamber (Wheeler et al., 2003; Dreschel et al., 2018), the Russian BIOS facilities (Gitelson et al., 2003), the Japanese Closed Ecology Experimental Facility (Nitta, 2005) and the Chinese Lunar Palace 1 (Fu et al., 2016).

**FIGURE 1:**
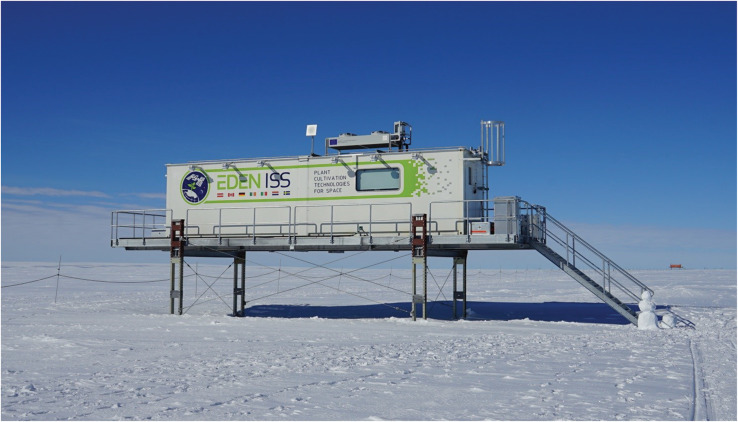
The EDEN ISS Mobile Test Facility in Antarctica around 400 m south of the German Neumayer Station III.

The EDEN ISS project is the newest space greenhouse analog project to test subsystems, technologies, operation procedures, plant health monitoring devices and plant cultivation for future space missions. The EDEN ISS Mobile Test Facility (MTF) (Figure 1) was setup in Antarctica to achieve these goals. This paper presents detailed values on food production (edible biomass) and inedible biomass production of the vegetable crops cultivated in the experimental phase in 2018.

The difference of the EDEN ISS MTF to the other facilities is its unique location. The MTF is positioned in the vicinity of the German Neumayer Station III in Antarctica. This continent offers several conditions which are favorable for space analog test campaigns (Bubenheim et al., 1994). While most of the other facilities were built to conduct research on humans living in a closed loop life support system, EDEN ISS focuses on cultivating plants in controlled conditions, testing the necessary hardware and investigating microbiology, food quality and safety aspects. Furthermore, most of the facilities mentioned earlier were built and operated in the 1980s–2000s and are no longer available, except for Lunar Palace 1 which was built only a few years ago and is still in operation. EDEN ISS also uses technologies which were not available in the past (e.g., LED lighting for plant cultivation).

The novel aspect of the EDEN ISS project is its approach to work with a compromise climate in which all crops are grown simultaneously. This is more realistic for near-term space greenhouses as compared to studies were each crop has its own optimized climate. Despite not having the optimal climate for each crop the food production of the MTF in the 2018 season was higher than expected. In 2018, for the first time a comprehensive set of measurements were performed in an analog space greenhouse. These measurements encompass the biomass production data presented in this paper, but also data on the microbial environment inside the greenhouse, the quality and safety of the produced food, the resources (e.g., carbon dioxide, nutrients, consumables) necessary to grow the crops, the amount of electrical energy and crewtime required and the acceptance of the food to the station crew. The biomass production dataset presented in this paper can be used to improve simulation models for space greenhouses. It can also be used for cultivar selection, because the dataset includes values on different cultivars (e.g., for lettuce and tomato) which is helpful to assess which cultivar should be grown in the next space greenhouse. The data is also a valuable contribution to the recently developed Crop Readiness Level evaluation method (Romeyn et al., 2019) for crop candidates for space greenhouses.

## Materials and Methods

### EDEN ISS Mobile Test Facility Infrastructure

**FIGURE 2:**
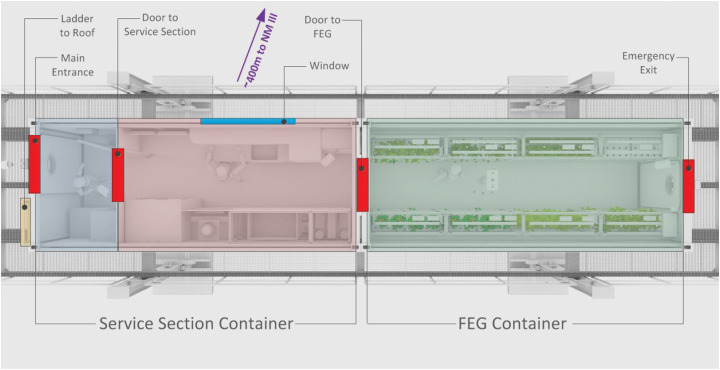
Schematic top-view of the MTF. The blue section indicates the cold porch. The red area is called service section and houses a work desk and almost all greenhouse subsystems. The green section is the main plant cultivation space, called the FEG.

The EDEN ISS MTF is located in the immediate vicinity of the Neumayer III Station which is operated by the German Alfred-Wegener Institute for Polar and Marine Research. The MTF was designed and built as an experimental facility for plant cultivation systems, allowing the test of essential technologies and production procedures for future long-duration human space missions (Zabel et al., 2015). Detailed system analysis was conducted by the consortium partners resulting in a solid design (Bamsey et al., 2014, 2016; Zabel et al., 2016b), including a complete risk assessment (Santos et al., 2016). The MTF consists of two customized 20 foot high cube shipping containers, which are placed on top of a raised platform located ∼400 m south of Neumayer III. The research facility can be subdivided into three distinct sections:

• Airlock/Cold-Porch (Blue section in Figure 2): A small room providing storage and a small air buffer to limit the entry of cold air when the main access door of the facility is used. This area is used for changing clothes. Furthermore, the main fresh water tank and the waste water tank are both located in the subfloor space of this section.
• Service Section (Red section in Figure 2): This section houses the primary control, atmosphere management, thermal control, power control and nutrient delivery systems of the MTF (Vrakking et al., 2017). Additionally, this section provides a working table including sink, trash bins, and storage for tools and consumables. In addition this section houses an independent rack-like plant cultivation system as part of the plant growth demonstrator for future deployment onboard the ISS (Boscheri et al., 2016, 2017).
• Future Exploration Greenhouse (FEG) (Green section in Figure 2): The main plant cultivation space of the MTF which includes multi-level plant growth racks operating in a controlled environment. The FEG is used to study plant cultivation and the related technologies for future planetary habitats (Zabel et al., 2017).

The technologies required to cultivate plants in a controlled environment are arranged in six different subsystems, which are briefly described in the following. Detailed information about the subsystems can be found in Zabel et al. (2017) and Vrakking et al. (2017).

1) The nutrient delivery subsystem adjusts the irrigation water’s pH and EC value. Depending on the plant type (leafy or fruit-building crop), the mixing computer provides a dedicated nutrient solution that is delivered directly to the roots. Eight high-pressure pumps spray a fine nutrient mist inside the root compartment of each plant tray.
2) The atmosphere management subsystem regulates the temperature, humidity, and CO_2_ concentration within the FEG. Furthermore, the air flow is filtered (particle filter, HEPA, and activated carbon filter) and the humidity condensate water is recovered and fed back to the fresh water tank.
3) The thermal control subsystem is used to remove excess heat from the MTF and to provide a cool fluid for condensation of the humidity produced by the plants.
4) The illumination control subsystem consists of 42 fluid-cooled LED fixtures integrated into the FEG. The light spectrum can freely be composed of red, blue, far-red, and white for each plant tray.
5) The power distribution subsystem provides electrical energy to all subsystems of the MTF. The electrical energy is generated in the Neumayer Station III and transmitted to the MTF.
6) The control and data handling subsystem consists of a set of independent programmable logic controllers which receive information from a wide range of sensors. Based on this information and defined program logics this subsystem controls all functions of the MTF. The control and data handling subsystem sends system telemetry to the mission control center in Bremen, Germany. Furthermore, every day a set of images taken from fixed positions inside the FEG is sent to the mission control center to allow remote experts observing plant development and to assist the on-site operator.

### Experiment Timeframe

**FIGURE 3:**
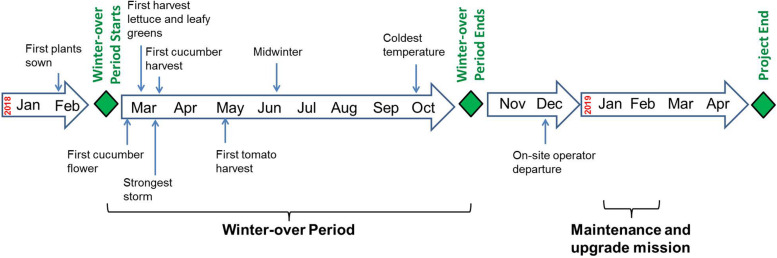
Timeline of the EDEN ISS experiment phase (early 2018 to early 2019).

The MTF arrived in Antarctica on the 3rd of January 2018. The deployment took around 5 weeks and was finished in early February (Schubert et al., 2018). The winter-over on-site operator remained in Antarctica for the following 10 months until December 2018. In the following chapters “experimental phase” refers to the period from mid-February 2018 to mid-November 2018. The timeline of the experiment phase is shown in Figure 3.

The first plants were sown on February 7th 2018. The winter-over period started with the departure of the last summer crew on February 18th. The first harvest of lettuce and leafy greens was on March 20th. The first cucumber harvest (29th of March) and first tomato harvest (16th of May) took place in the weeks that followed. The coldest temperature of the season was recorded in the morning of the 8th of October 2018 with being −43.5°C. The winter-over period ended with the arrival of the first summer crew on November 2nd. The nominal operation phase of the MTF ended with the final harvest which took place on November 20th. The EDEN ISS winter-over operator departed a few days before Christmas 2018.

### Crop Species

The EDEN ISS project team established a crop selection methodology in order to select crop species for the experiment campaign in Antarctica (Dueck et al., 2016). The focus of the project was on fresh pick-and-eat vegetables. Consequently, the list of cultivated crop species includes various varieties of lettuce and leafy greens accompanied by some fruit crops (e.g., tomato and cucumber).

The cultivated crops are organized in the five categories lettuce, leafy greens, herbs, fruit crops and tuber crops. The following crop varieties were cultivated:

• Lettuce: Batavia, Expertise, Outredgeous, Waldmann’s Green
• Leafy greens: Two varieties of red mustard, swiss chard, arugula, mizuna
• Herbs: Basil, chives, parsley
• Fruit crops: Red dwarf tomato, orange dwarf tomato, cucumber (parthenocarpic, beit alpha)
• Tuber crops: Two varieties of radish, kohlrabi
• Miscellaneous crops: Two varieties of indeterminate tomato, two varieties of pepper, cilantro, mint, lemon balm, celery, strawberry.

Additional information (e.g., seed supplier) about the cultivated crop species can be found in Supplementary Material.

### Plant Treatment

Most of the crop species were sown in rockwool blocks of 2 × 2 × 4 cm (L × W × H), with the exception being parsley, chives and arugula. Those crops were sown directly in the cultivation trays on mats consisting of recycled cotton fibers. The crops grown in rockwool blocks were first put into a nursery tray for 10–20 days depending on the species. Small amounts of nutrient solution were added manually to this tray in order to keep the rockwool blocks moist. Following the period in the nursery tray, the young plants were moved to the plant cultivation trays for maturation. Some crop species (cucumber, tomato, and pepper) required regular pruning of excess side shoots and leaves.

Two different methods for cultivation/harvest were used. Batch cultivation means that the whole plant was harvested when the plant reached a certain age. This technique was used for the lettuce varieties, radishes, and kohlrabi. Spread harvest means that only parts of the plant (leaves, fruits) were harvested allowing the plant to continue growing. All fruit crops, all herbs and all leafy greens were harvested this way.

Dates for sowing, transfer, pruning, and harvest events per cultivation tray were tracked continuously. Plant density can be determined from the type of cultivation tray which was used. Table 1 summarizes information regarding the plant treatment for each crop. Upon harvest fresh edible and inedible biomass was measured. The latter was measured separately for roots and stems/leaves. Sometimes plant material was dried using lyophilization in order to determine the dry biomass ratio which is the ratio of the dried biomass weight to the original fresh biomass weight. Drying plant material was limited due to the sizing of the equipment and due to the fact that dried material was required for each crop species.

**TABLE 1:** Summary of plant treatment information.

Crop	Crop density	Crop density		Harvest
name	(plants/tray)	(plants/m^2^)	Pruning	type
Batavia	6	18.3	n.a.	Batch
Expertise	6	18.3	n.a.	Batch
Outredgeous	6	18.3	n.a.	Batch
Waldmann’s Green	6	18.3	n.a.	Batch
Red Giant	20	61.0	n.a.	Spread
Frizzy Lizzy	20	61.0	n.a.	Spread
Swiss Chard	12	36.6	n.a.	Spread
Arugula	196	594.5	n.a.	Batch
Mizuna	12	36.6	n.a.	Spread
Basil Dolly	20	61.0	Shortening of shoots when getting to close to LED lamps.	Spread
Parsley	∼100	304.9	n.a.	Spread
Chives	∼200	609.8	n.a.	Spread
Tomato F1 3496B	4	12.2	Periodic removal of withered leaves. Removal of withered side shoots after harvest period to encourage plant to regrow new side shoots.	Spread
Tomato F1 1202	4	12.2	Periodic removal of withered leaves. Removal of withered side shoots after harvest period to encourage plant to regrow new side shoots.	Spread
Cucumber Picowell	2	6.1	2 shoots/stems per plant. Periodic removal of excess side shoots and old leaves.	Spread
Radish Raxe	36	109.8	n.a.	Batch
Radish Lennox	36	109.8	n.a.	Batch
Kohlrabi	5–6	15.2–18.3	Removal of single leaves when those would block light neighboring trays.	Batch

Plant development was monitored by several cameras. From each plant cultivation tray, one photo from the top and one photo from the side were taken every day and send to a FTP server where all project partners could access the images. This way the horticulture scientists in the project team could advise the on-site operator on improvements for the cultivation of the crops. An image processing algorithm checked the photos automatically to detect issues with plant development. A multi-wavelength imaging system was setup in two positions to test whether this system can detect plant stress during growth (Zeidler et al., 2019).

### Environmental Conditions During Plant Cultivation

#### Irrigation

High pressure pumps in the FEG feed nutrient solution from the tanks to the plant cultivation trays via a hybrid aeroponic and nutrient film technique (Vrakking et al., 2017; Zabel et al., 2017). The solution was injected into the root zone via misting nozzles and the run-off served as a nutrient film once the roots had developed sufficiently. The irrigation schedule for the plants was a 30 s misting period every 6 min. For the initial germination phase, the on-site operator manually supplied nutrient solution to the germination tray.

Two different nutrient solutions were provided to the crops, depending on their classification as either a leafy crop or a fruit crop. The two solutions were automatically mixed together in bulk solution tanks using deionized water and nutrients from concentrated stock solution bottles. The expected (initial) nutrient concentrations in the bulk solutions, based on the recipes developed for the project, can be seen in Table 2. The pH value of the solutions was managed by utilization of acid (1.25% Nitric acid) and base (1% Potassium hydroxide) stock solutions.

**TABLE 2:** Nutrient concentration in 100 L bulk solution in NDS tanks during the experiment phase.

Nutrient	Leafy crop solution	Fruit crop solution
compound	concentrations	concentrations
NH4	0.122 mol	0.226 mol
K	1.028 mol	1.503 mol/*1.378 mol*
Ca	0.419 mol	0.597 mol/*0.711 mol*
Mg	0.135 mol	0.226 mol
NO3	1.785 mol	2.347 mol/*2.450 mol*
Cl	0.068 mol	0.104 mol
SO4	0.109 mol	0.332 mol
P	0.189 mol	0.267 mol
Fe	3.795 mmol	5.161 mmol
Mn	0.189 mmol	2.059 mmol
Zn	0.244 mmol	0.825 mmol
B	2.840 mmol	4.321 mmol
Cu	0.068 mmol	0.164 mmol
Mo	0.041 mmol	0.103 mmol

*Note that the composition of the fruit crop solution was adapted during the mission. Adjusted values are shown in italic (The adjustment was necessary because a calcium deficit could be observed on the tomato plants, which most likely was the result of a bad K:Ca ratio).*

Based on the plant development observed throughout the operations phase, and in communication with remote experts in Europe, it was decided part way throughout the mission to adjust the composition for the fruit crop nutrient solution to include more calcium and to reduce the amount of potassium slightly. The concrete values are given in Table 2.

There are only two main points of control for the Nutrient Delivery Subsystem (NDS), pH and EC. Both of these operated as expected throughout the first season of plant production. After the initial setup and testing of the growing systems in the beginning 30 days of operation, pH control was excellent. Any deviations from the set point were due to easily diagnosed technical issues (e.g., broken connectors on the acid delivery supply lines) and pH was never beyond a level suitable for plant growth. Tank 1 followed a higher control level than that of tank 2, and this was due to a programmed offset within the control software. Tank 1 averaged pH 6.06 ± 0.18 and tank 2 averaged 5.91 ± 0.12 over the entire growing period.

EC monitoring results were similar to that of pH. Control was excellent after the initial 30 day setup period. Each nutrient tank had a different EC set point, and control was tight with 2.21 ± 0.13 mS/cm and 3.49 ± 0.17 mS/cm in tanks 1 and 2, respectively. At no time did EC deviate outside of a range amenable to plant growth and productivity.

Also monitored but not controlled was the nutrient solution temperature in the main nutrient tanks. Tank temperature followed that of the well-controlled room temperature and was quite stable with 19.90 ± 0.74°C in tank 1 and 19.97 ± 0.78°C in tank 2.

As continuous determination of and control over individual ion concentrations was not possible, it was decided to periodically empty the bulk nutrient solution tanks and start with a new mixture. For tank 1 this procedure was done approximately once every 3 months, whereas for tank 2 the exchange was done about once every 2 months.

#### Illumination

A detailed description of the illumination subsystem is provided by Zabel et al. (2016b). Each plant cultivation tray has its own LED lamp which can be independently controlled in terms of light spectrum and light intensity. The LED lamps are of the model LX601 by the Swedish company Heliospectra which were modified with a liquid cooling system instead of the air cooling system. This way the light settings for each tray can be adjusted to the crop species and plant maturation stage. The photoperiod inside the FEG consisted of 15 h of full illumination per day and 1 h of reduced light intensity (50% of nominal intensity) before and after the full illumination period. Consequently, the dark period was 7 h per day. The light spectrum mainly consisted of blue (∼450 nm) and red (∼650 nm) light and small portions of other wavelengths. The light intensity varied between 300 and 600 μmol/(m^2*^s) at canopy level of a mature plant, depending on crop species and plant age. The following light intensities were measured for the different crops:

1. All four lettuce varieties: 330 μmol/(m^2*^s) at 16 cm height.
2. Red mustard, swiss chard, mizuna, basil, chives, parsley: 330 μmol/(m^2*^s) at 16 cm height.
3. Radish, arugula: 600 μmol/(m^2*^s) at 16 cm height.
4. Tomato, Kohlrabi, pepper: 300–400 μmol/(m^2*^s) at 16 cm height.
5. Cucumber: > 500 μmol/(m^2*^s) at top of canopy.

#### Atmosphere

**FIGURE 4:**
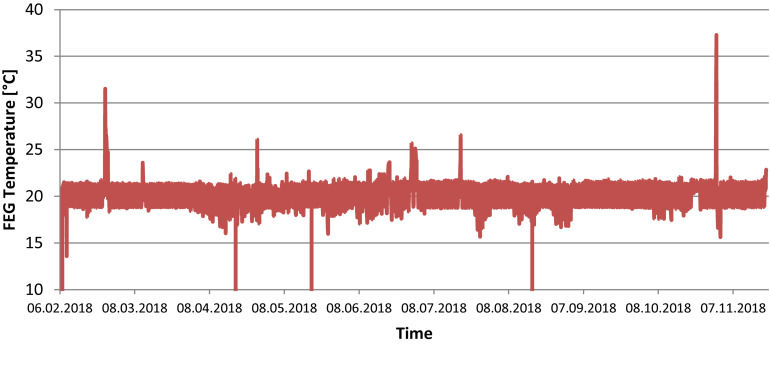
FEG air temperature during the experimental phase (one data point per minute).

**FIGURE 5:**
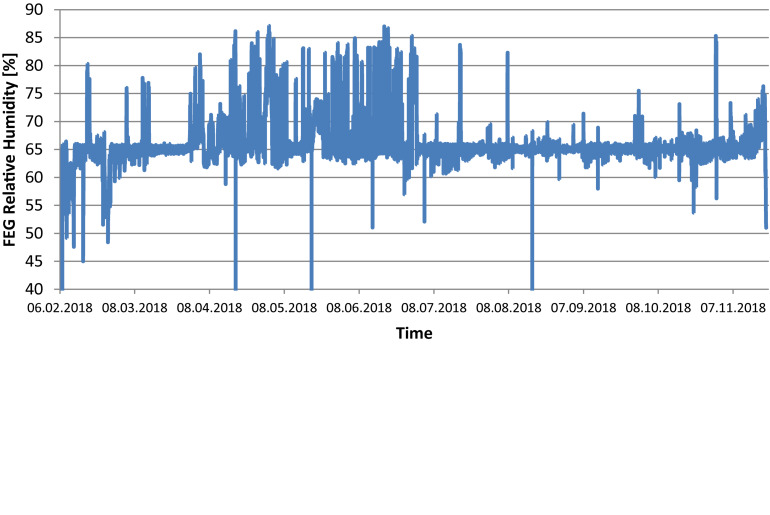
FEG relative humidity during the experimental phase (one data point per minute).

**FIGURE 6:**
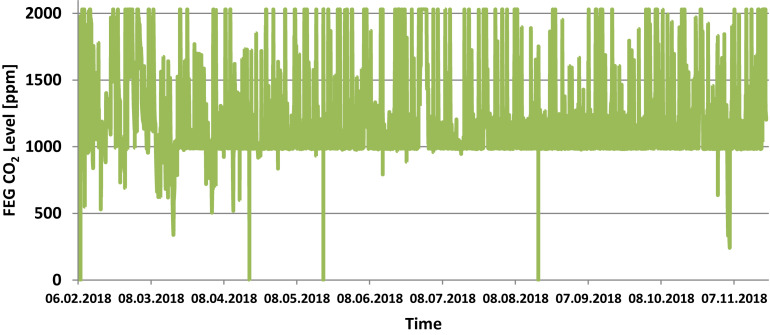
FEG atmospheric CO_2_ level during the experiment phase (one data point per minute). Note that the sensor used had an upper limit of 2000 ppm. This value was exceeded frequently due to the presence of the on-site operator.

The temperature set points inside the FEG were 21°C during the photoperiod and 19°C during the dark period. Relative humidity was set to 65% and CO_2_ concentration to 1000 ppm. Figures 4–6 show the actual values measured within the FEG throughout the first year of operations. In general it can be seen that the temperature was maintained at the set points, fluctuating between 21 degrees during the photoperiod and 19 degrees during the dark period.

During the Antarctic winter season, at very low external temperatures, the facility failed to maintain temperature during the dark period, resulting in temperature drops down to 16°C. Some off-nominal events with the thermal control subsystem resulted in temperature increases beyond the 21°C set point. Furthermore, a number of measurements are erroneous (showing 0°C) due to communication issues in the command and data handling subsystem.

The relative humidity within the FEG showed larger deviations from the set point of 65%, in particular between April and July the relative humidity would go as high as 86% for periods of a few hours. This was the result of issues with the condensate recovery design, which had to be mitigated by adjusting flow rates and coolant temperatures within the thermal control subsystem and fine-tuning the control logic. Following the troubleshooting phase, and implementing countermeasures, the relative humidity control throughout the later phase of the experiment phase was significantly improved.

The CO_2_ concentration within the FEG was almost always higher than the set point of 1000 ppm, due to the fact that the on-site operator frequently (almost every day) worked within the greenhouse, emitting CO_2_, and there is no CO_2_ removal system implemented within the facility. Depending on the time (e.g., 1–4 h) spent inside the FEG, the CO_2_ concentration went up to ∼2000 ppm or even ∼4000 ppm. Normally the plants in the FEG would need around 1 day to consume enough CO_2_ to bring the concentration back down to the set point of 1000 ppm.

A desired maximum ethylene concentration of 15 ppb was defined for the greenhouse, with up to 100 ppb allowed for durations of no more than 30 min. An activated carbon filter was implemented into the atmosphere management subsystem to remove ethylene. However, no sensor was installed to provide measurements of the actual concentration within the facility.

## Results

### Overview

The FEG produced a total of around 268 kg of fresh edible biomass. Most of this food was consumed by the 10 person strong winter-over crew. Small amounts were set aside to perform a wide range of measurements (e.g., dry weight ratio, nitrate content). The fruit crops produced by far the most food (105.4 kg) followed by the lettuce (56.4 kg), leafy greens (49.1 kg), tuber crops (26.8 kg), herbs (12.2 kg), and miscellaneous crops (18.4 kg).

The evaluation of the inedible biomass was rather complicated, because some of the inedible biomass was harvested wet (e.g., roots and rock wool), some fresh (e.g., radish leaves), and some dry (e.g., withered tomato leaves). Combining all three values into a single figure for all crops was challenging, because the dry biomass ratio was required. In total roughly 11.2 kg of dry inedible biomass (excluding misc. crops) was harvested.

The following chapters give a detailed overview of the biomass production of each crop species.

### Lettuce

In total 18 cycles of lettuce cultivation were performed in the experiment phase. Between 11 and 15, depending on the lettuce cultivars, of these cycles are valid for evaluation. The remaining cycles have been excluded from evaluation due to various reasons (e.g., extensive sampling of plant material). The four lettuce varieties were always sown and harvested at the same time and cultivated in trays located in the same rack and with the same environmental conditions. Nevertheless, the biomass production varied greatly among the crop varieties, as can be seen in Table 3. Waldmann’s Green clearly produced the most biomass per tray, followed by Expertise, Outredgeous, and Batavia. Data on the dry weight ratio for edible and inedible biomass can be found in Table 4 and dry inedible biomass production in Table 5.

**TABLE 3:** Lettuce edible fresh weight production values.

			Edible fresh	Time normalized	Edible fresh
		Average	weight per	edible fresh weight	weight per
	Cycles for	cultivation	cultivation area	per cultivation area	cultivation volume
Crop name	evaluation	cycle length (d)	(kg/m^2^)	(kg/m^2^/d)	(kg/m^3^)
Batavia	15	38.0	1.56 ± 0.12 (0.98–2.46)	0.043 ± 0.002 (0.028–0.059)	1.77–4.43
Expertise	15	38.0	2.38 ± 0.16 (1.50–3.50)	0.065 ± 0.003 (0.043–0.084)	2.70–6.31
Outredgeous	14	37.9	2.10 ± 0.15 (1.39–2.65)	0.058 ± 0.003 (0.038–0.072)	2.50–4.77
Waldmann’s Green	11	37.9	2.77 ± 0.30 (1.42–4.36)	0.080 ± 0.007 (0.037–0.115)	2.56–7.86

*Mean ± standard error is given.*

**TABLE 4:** Dry weight (DW) ratio values for edible and inedible biomass.

	Edible	Inedible	Inedible
	biomass	biomass DW	biomass DW
Crop	DW ratio	ratio (roots)	ratio (stems/leaves)
name	(%)	(%)	(%)
Batavia	5.79 ± 0.30	3.90 ± 1.26	n.a.
	*N* = 12	*N* = 3	
Expertise	6.09 ± 0.34	4.57 ± 1.18	n.a.
	*N* = 12	*N* = 3	
Outredgeous	6.61 ± 0.35	4.82 ± 0.92	n.a.
	*N* = 12	*N* = 3	
Waldmann’s Green	6.35 ± 0.44	2.69 ± 0.29	n.a.
	*N* = 12	*N* = 3	
Red Giant	6.09 ± 0.13	4.07	6.43
	*N* = 17	*N* = 1	*N* = 1
Frizzy Lizzy	6.04 ± 0.13	4.07	5.53
	*N* = 17	*N* = 1	*N* = 1
Swiss Chard	7.16 ± 0.12	10.08 ± 3.09	6.26 ± 1.74
	*N* = 10	*N* = 3	*N* = 4
Arugula	6.52 ± 0.40	4.24 ± 0.21	n.a.
	*N* = 16	*N* = 6	
Mizuna	6.37 ± 0.49	Not measured	Not measured
	*N* = 3		
Basil Dolly	8.64 ± 0.31	3.18 ± 1.03	6.02 ± 2.48
	*N* = 19	*N* = 2	*N* = 2
Parsley	10.93 ± 0.09	7.15	Not measured
	*N* = 10	*N* = 1	
Chives	8.84 ± 0.10	7.15	Not measured
	*N* = 10	*N* = 1	
Tomato F1 3496B	12.66 ± 0.29	10.21 ± 1.16	25.43 ± 1.15
	*N* = 13	*N* = 4	*N* = 10
Tomato F1 1202	13.36 ± 0.75	9.63 ± 1.04	28.35 ± 3.94
	*N* = 13	*N* = 4	*N* = 10
Cucumber Picowell	4.43 ± 0.13	3.20 ± 0.39	16.51 ± 0.88
	*N* = 18	*N* = 4	*N* = 11
Radish Raxe	5.79 ± 0.17	0.59 ± 0.06	8.09 ± 0.35
	*N* = 15	*N* = 3	*N* = 18
Radish Lennox	Not measured	Not measured	Not measured
Kohlrabi	7.17 ± 0.35	3.78 ± 0.51	Leaves:
	*N* = 5	*N* = 4	9.49 ± 0.26
			*N* = 8
			Skin:
			7.97 ± 0.28
			*N* = 6

*Mean ± standard error is given.*

**TABLE 5:** Inedible biomass production overview.

Crop	Roots	Stems and
name	DW	leaves DW
Batavia	8.9–34.2 g/m^2^ (*N* = 3)	n.a.
Expertise	20.5–37.9 g/m^2^ (*N* = 3)	n.a.
Outredgeous	21.1–38.5 g/m^2^ (*N* = 3)	n.a.
Waldmann’s Green	11.3–22.9 g/m^2^ (*N* = 3)	n.a.
Red Giant	42.0 g/m^2^ (*N* = 1)	75.6 g/m^2^ (*N* = 1)
Frizzy Lizzy	42.0 g/m^2^ (*N* = 1)	20.8 g/m^2^ (*N* = 1)
Swiss Chard	21.8–47.0 g/m^2^ (*N* = 2)	81.4–94.4 g/m^2^ (*N* = 2)
Arugula (initial light intensity)	Not measured	n.a.
Arugula (higher light intensity)	21.3–58.8 g/m^2^ (*N* = 6)	n.a.
Mizuna	Not measured	Not measured
Basil Dolly	76.5–127.1 g/m^2^ (*N* = 2)	179.3–252.1 g/m^2^ (*N* = 2)
Parsley	146.3 g/m^2^ (*N* = 1)	113.4 g/m^2^ (*N* = 1)
Chives	146.3 g/m^2^ (*N* = 1)	1033.5 g/m^2^ (*N* = 1)
Tomato F1 3496B	1411–1713 g/m^2^ (*N* = 2)	Erroneous measurement
Tomato F1 1202	1153–1567 g/m^2^ (*N* = 2)	Erroneous measurement
Cucumber Picowell	93.8–133.5 g/m^2^ (*N* = 4)	Erroneous measurement
Radish Raxe	96.4–99.4 g/m^2^ (*N* = 3)	89.0–123.5 g/m^2^ (*N* = 2)
Radish Lennox	Not measured	Not measured
Kohlrabi	27.8–51.0 g/m^2^ (*N* = 3)	198.4–253.3 g/m^2^ (*N* = 2)

For all lettuce varieties a large variance between batches could be observed and this is also visible in the values of the standard error. There was a decline in biomass harvest roughly in the mid of the season. The productivity of the batches harvested in June and July was less than half of the maximum value.

### Leafy Greens

Five different leafy greens were cultivated. Arugula was harvested in batches, while the other leafy greens (red mustard, swiss chard, and mizuna) were spread harvested. Furthermore, arugula was cultivated with two different light settings. Table 6 shows the biomass production values of all leafy greens. Mizuna and arugula (high light intensity, 600 μmol/(m^2*^s) at 16 cm height) performed best followed by Swiss chard, both red mustard varieties and arugula (low light intensity, 330 μmol/(m^2*^s) at 16 cm height) which all had a similar output. Due to the batch harvesting of arugula and the availability of two trays for simultaneous cultivation 17 cycles are available for evaluation. For the other leafy greens, only between 1 and 3 cycles, depending on the cultivar, of data are available. Data on the dry weight ratio for edible and inedible biomass can be found in Table 4 and dry inedible biomass production in Table 5.

**TABLE 6:** Leafy greens edible fresh weight production values.

			Edible fresh	Time normalized	Edible fresh
		Average	weight per	edible fresh weight	weight per cultivation
	Cycles for	cultivation	cultivation area	per cultivation area	volume
Crop name	evaluation	cycle length (d)	(kg/m^2^)	(kg/m^2^/d)	(kg/m^3^)
Arugula (initial light intensity, 330 μmol/(m^2^*s) at 16 cm height)	9	24.2	3.05 ± 0.27 (1.87–4.34)	0.111 ± 0.007 (0.078–0.140)	3.37–7.82
Arugula (higher light intensity, 600 μmol/(m^2^*s) at 16 cm height)	8	29.0	5.49 ± 0.40 (4.13–6.90)	0.188 ± 0.011 (0.153–0.222)	7.44–12.43
Swiss Chard	3	90.0	9.26 ± 1.13 (7.35–11.28)	0.102 ± 0.010 (0.088–0.121)	13.24–20.32
Red Giant	3	82.7	10.78 ± 0.52 (8.73–11.96)	0.130 ± 0.004 (0.106–0.145)	15.72–21.54
Frizzy Lizzy	3	82.7	8.93 ± 0.48 (7.11–10.37)	0.107 ± 0.005 (0.086–0.125)	12.80–18.70
Mizuna	1	119.0	23.11	0.194	41.64

*Mean ± standard error is given.*

### Herbs

Basil, chives, and parsley were cultivated during the experiment phase. All herbs were spread harvested. Parsley and chives were grown for almost the complete duration of the experiment phase (266 out of 286 days). Basil had to be removed from the trays and sown anew regularly, because the plants grew rapidly, reaching up to the LED lamps after several weeks. Four cycles of basil were grown of which two are suitable for data evaluation. The other two had to be excluded because the first cycle used an inappropriate growing procedure and the last cycle was too short. The three herbs have a similar production rate of edible biomass with parsley and basil being slightly ahead of chives. The biomass production data for the herbs can be found in Table 7. Data on the dry weight ratio for edible and inedible biomass can be found in Table 4 and dry inedible biomass production in Table 5.

**TABLE 7:** Herbs edible fresh weight production values.

		Average	Edible fresh	Time normalized	Edible fresh
		cultivation	weight per	edible fresh weight	weight per
	Cycles for	cycle	cultivation	per cultivation	cultivation
Crop name	evaluation	length (d)	area (kg/m^2^)	area (kg/m^2^/d)	volume (kg/m^3^)
Basil	2	121.0	7.30 ± 0.93 (6.37–8.22)	0.060 ± 0.008 (0.052–0.069)	11.48–14.81
Chives	1	266.0	13.97	0.053	25.17
Parsley	1	266.0	16.46	0.062	29.66

*Mean ± standard error is given.*

### Fruit Crops

Fruit crops produced the most edible biomass during the experiment phase. Especially the cucumber showed an exceptional productivity of more than 100 g/(tray^∗^d) and consequently contributed the most edible biomass of all cultivated crop species. The two dwarf tomato varieties show a similar productivity, with the orange tomato being slightly lower than the red one. The tomato plants were grown in a single cycle lasting the full experimental phase, while the cucumber plants were grown in two cycles. The biomass production data for the fruit crops can be found in Table 8. Data on the dry weight ratio for edible and inedible biomass can be found in Table 4 and dry inedible biomass production in Table 5.

**TABLE 8:** Fruit crops edible fresh weight production values.

		Average	Average	Edible fresh	Time normalized	Edible fresh
		cultivation	fruit per	weight per	edible fresh weight	weight per
	Cycles for	cycle	cycle per	cultivation	per cultivation	cultivation
Crop name	evaluation	length (d)	tray	area (kg/m^2^)	area (kg/m^2^/d)	volume (kg/m^3^)
Tomato F1 3689B	2	286.0	994	13.06 ± 0.00 (13.06–13.07)	0.046 ± 0.000 (0.046–0.046)	11.82–11.83
Tomato F1 1202	2	286.0	1372	14.90 ± 1.84 (13.06–16.78)	0.052 ± 0.006 (0.046–0.059)	11.82–15.19
Cucumber Picowell	4	161.0	208	50.88 ± 4.38 (41.70–59.98)	0.321 ± 0.041 (0.241–0.403)	18.45–26.54

*Mean ± standard error is given.*

### Tuber Crops

Tuber crops (radish and kohlrabi) were harvested in batches. 20 batches of radishes were grown during the experiment phase, 10 of each variety. Of those 20 batches, 19 were suitable for evaluation. One batch had to be excluded from evaluation due to extensive sampling of plant material for microbial and matter analyses (e.g., nitrate content, antioxidants). Furthermore, seven batches of kohlrabi were grown. Kohlrabi produced more biomass per cultivation area and time normalized than radish. The biomass production data for the fruit crops can be found in Table 9. Data on the dry weight ratio for edible and inedible biomass can be found in Table 4 and dry inedible biomass production in Table 5.

**TABLE 9:** Tuber crops edible fresh weight production values.

		Average	Edible fresh	Time normalized	Edible fresh
		cultivation	weight per	edible fresh weight	weight per
	Cycles for	cycle	cultivation	per cultivation	cultivation
Crop name	evaluation	length (d)	area (kg/m^2^)	area (kg/m^2^/d)	volume (kg/m^3^)
Radish Raxe	10	22.6	1.82 ± 0.24 (1.10–3.21)	0.078 ± 0.009 (0.044–0.119)	1.98–5.78
Radish Lennox	9	23.0	1.33 ± 0.06 (1.07–1.68)	0.059 ± 0.002 (0.045–0.068)	1.93–3.03
Kohlrabi	6	58.71	8.11 ± 0.81 (5.74–10.58)	0.141 ± 0.008 (0.113–0.165)	10.34–19.06

*Mean ± standard error is given.*

## Discussion

During the 286-day operational phase in 2018 the EDEN ISS MTF produced 268 kg of fresh edible biomass, which is a good result for the first year of operation. The production rate was 21.44 kg/m^2^. This results in a time normalized production rate of 0.075 kg/(m^2*^d). The pepper plants did only produce small amounts of fruit in the 2018 EDEN ISS season, but took up 11% of the cultivation area. When correcting the overall production of EDEN ISS by removing the pepper plants from the calculation the edible fresh biomass production rate increases to 0.089 kg/(m^2*^d). The South Pole Food Growth Chamber (SPFGC), an indoor plant cultivation room at the American South Pole Station (Patterson et al., 2008), had a production rate of 0.130 kg/(m^2*^d) in 2006 (Patterson et al., 2012). The SPGFC mainly produced lettuce (32% of total fresh edible biomass) and cucumber (41%) and only small amounts of herbs (6%), tomato (4%), and other crops (17%). Whereas the distribution in EDEN ISS in 2018 was 21% lettuce, 18% leafy greens, 25% cucumber, 5% herbs, 14% tomato, 10% tuber vegetables and 7% other crops. Since cucumber have the highest production rate per unit area and time, the higher ratio of cucumber in the SPFGC harvest can explain the better overall production rate of fresh edible biomass compared to EDEN ISS to some degree.

Plant cultivation experiments have been conducted by an EDEN ISS project partner in advance of the Antarctic experimental campaign (Meinen et al., 2018). When comparing the results from Antarctica with the experiments conducted in plant growth chambers in Europe, the yield per unit time and cultivation area of lettuce was higher in Antarctica than in the experiments in Europe. The yield of the red mustard frizzy lizzy, Swiss chard, parsley and chives was better in the plant growth chambers in Europe than in Antarctica, but the plant density in those experiments was much higher.

The yield of lettuce was better than (Richards et al., 2004b; Edney et al., 2006) or equal to (Richards et al., 2004a) some other experiments, but only half as good as the values achieved by the BPC (Wheeler et al., 2008). No reliable reference data could be found for the leafy greens mizuna and the red mustard red giant. This is also the issue with the cultivated herbs basil, parsley and chives for which the only comparison that could be made was with the preparatory experiments of the project (Meinen et al., 2018). The dwarf tomato yield was basically equal to experiments with similar cultivars (Spencer et al., 2019; Wang et al., 2019), but smaller compared to the BPC results (Wheeler et al., 2008) which were most likely done with normal sized tomato crops. The yield of the EDEN ISS cucumber cultivation was better compared to the experiments with this crop in Lunar Palace 1 (Fu et al., 2016). The comparisons between the results from the experiment campaign in Antarctica in 2018 to other experiments are summarized in Table 10.

**TABLE 10:** Comparison of EDEN ISS time normalized edible fresh biomass production rate to the results of experiments by other scientists.

Crop name	EDEN ISS [kg/(m^2^*d)]	Experiments by other scientists [kg/(m^2^*d)]
Batavia	0.043	Meinen et al. (2018): 0.033; same environmental conditions.
Expertise	0.065	Meinen et al. (2018): 0.051; same environmental conditions.
Outredgeous	0.058	Meinen et al. (2018): 0.040; same environmental conditions. Richards et al. (2004b): 0.036^2^; 25°C, 65% rh, 1200 ppm CO_2_, 300 μmol/(m^2^*s), 16 h photoperiod.
Waldmann’s Green	0.080	Wheeler et al. (2008): 0.161^2^; 23°C, 65–75% rh, 1000–1200 ppm CO_2_, 280–336 μmol/(m^2^*s), 16 h photoperiod.
Other lettuce types	n.a.	Edney et al. (2006), Flandria type: 0.029^2^; 22°C, 50% rh 1200 ppm CO_2_, 300 μmol/(m^2^*s), 16 h photoperiod. Richards et al. (2004a), Flandria type: 0.063; 25°C, 50% rh, 1200 ppm CO_2_, 300 μmol/(m^2^*s), 16 h photoperiod.
Red Giant	0.130	No reference literature found
Frizzy Lizzy	0.107	Meinen et al. (2018): 0.322; same environmental conditions, but 10 times higher plant density.
Swiss Chard	0.102	Meinen et al. (2018): 0.377; same environmental conditions, but 10 times higher plant density.
Arugula (initial light intensity)	0.111	
Arugula (higher light intensity)	0.188	Meinen et al. (2018): 0.162; same environmental conditions.
Mizuna	0.194	No reference literature found
Other leafy greens	n.a.	Fu et al. (2016)^1^: 0.100; 500 μmol/(m^2^*s), no values for temperature, rh, CO_2_ and photoperiod given.
Basil Dolly	0.060	No reference literature found
Parsley	0.062	Meinen et al. (2018): 0.143; same environmental conditions, but 3 times higher plant density.
Chives	0.053	Meinen et al. (2018): 0.052; same environmental conditions.
Tomato F1 3496B	0.046	Wang et al. (2019): 0.048; 25°C, 70% rh, 400 ppm CO_2_, 250 μmol/(m^2^*s), 14 h photoperiod. Spencer et al. (2019): 0.049; 22°C, 60% rh, 1507 ppm CO_2_, 324 μmol/(m^2^*s), 16 h photoperiod. Masuda et al. (2005): 0.028; 330 μmol/(m^2^*s), no values for temperature, rh, CO_2_ and photoperiod given. Wheeler et al. (2008): 0.075^2^; 26°C, 65–75% rh, 1000–1200 ppm CO_2_, 549–893 μmol/(m^2^*s), 12 h photoperiod.
Tomato F1 1202	0.052	
Cucumber Picowell	0.321	Fu et al. (2016): 0.228^3^; 500 μmol/(m^2^*s), no values for temperature, rh, CO_2_ and photoperiod given.
Radish Raxe	0.078	Meinen et al. (2018): 0.058; same environmental conditions. Edney et al. (2006): 0.203^4^; 22°C, 50% rh 1200 ppm CO_2_, 300 μmol/(m^2^*s), 16 h photoperiod.
Radish Lennox	0.059	
Kohlrabi	0.141	Masuda et al. (2005): 0.121; 330 μmol/(m^2^*s), no values for temperature, rh, CO_2_ and photoperiod given.

*^1^Reference lists dry mass production rate, which was converted assuming 6.0% dry matter content of fresh biomass. The dry mass content is based on EDEN ISS measurements. ^2^Reference lists dry mass production rate, which was converted assuming 13% dry matter content of fresh biomass. The dry mass content is based on EDEN ISS measurements. ^3^Reference lists dry mass production rate, which was converted assuming 4.4% dry matter content of fresh biomass. The dry mass content is based on EDEN ISS measurements. ^4^Reference lists dry mass production rate, which was converted assuming 5.8% dry matter content of fresh biomass. The dry mass content is based on EDEN ISS measurements.*

The FAOSTAT database, maintained by the United Nations Food Agriculture Organization, is a collection of yield values for a variety of commercially grown crops. This database can be filtered by crops and countries or regions of interest. Three crop categories similar to the EDEN ISS are found in the database: cucumbers/gherkins, tomatoes and lettuce/chicory. When looking on the FAOSTAT data for 2017 and setting the country to the Netherlands, the most effective producer of vegetables, one gets a yearly production of 68.97, 50.84, and 3.11 kg/m^2^ for cucumbers/gherkins, tomatoes and lettuce/chicory, respectively. The EDEN ISS values for the 2018 season converted to a yearly production are 122.99 kg/m^2^ cucumbers, 19.70 kg/m^2^ tomatoes and 36.11 kg/m^2^ lettuce. When comparing the values EDEN ISS production is much higher for cucumbers and lettuce, but only around 40% for tomatoes. The difference in tomato production is most likely caused by the decision to grow less-effective dwarf tomato cultivars instead of normal sized high-productive tomatoes, while the much higher yield of cucumbers and lettuce can be explained by the absence of seasonal temperature and illumination changes which affect conventional greenhouse farming.

## Summary

During the 9 month long experiment campaign of the international EDEN ISS space greenhouse analog project in Antarctica a wide range of vegetables were cultivated. These crop species are also candidates for greenhouses on future human spaceflight missions. The plants were cultivated in a closed, controlled environment using an aeroponic system and LED illumination. The on-site operator in 2018 harvested more than 268 kg of fresh food from the 12.5 m^2^ cultivation area of the EDEN ISS greenhouse. A description of the cultivation conditions is part of this paper. Detailed production values (edible and inedible biomass) for each crop species are shown in this paper as well as dry biomass ratios. Comparisons of the EDEN ISS yield from 2018 to other experiments are made.

The EDEN ISS MTF is the newest and most state-of-the-art space greenhouse analog experiment currently ongoing. The dataset presented in this paper can be of value to compare future experiments to and also for simulation and modeling efforts of space greenhouses. Furthermore, this first EDEN ISS biomass production data set states the beginning of a series of experimental seasons in Antarctica, which will continuously be recorded and published over the next years. One unique aspect of this research was the cultivation of all crops together in the same space under the same conditions. Although, this means that the conditions were not optimal for each cultivar, this is closer to how crop cultivation is going to be done in near-term space greenhouses, in which environmentally separated compartments for each crop are too costly and technically complex. This is in opposition to most laboratory experiments, in which crops are grown under optimal conditions.

## Data Availability Statement

The datasets generated for this study are available on request to the corresponding author.

## Author Contributions

PZ was the on-site operator of the EDEN ISS greenhouse in Antarctica during the experiment campaign, which means he was responsible for the plant cultivation and measurements, also worked on the materials and method part of the manuscript (section Materials and Methods), and on the data evaluation (section Results and Discussion). CZ, VV, MD, and DS were assisting the experiments from the mission control centre in Germany providing technical support and horticulture expertise. VV and DS provided input on the materials and method part of the manuscript (section Materials and Methods). DS was the project manager of EDEN ISS.

## Conflict of Interest

The authors declare that the research was conducted in the absence of any commercial or financial relationships that could be construed as a potential conflict of interest.
