# Supplementary material for: Biomass Production of the EDEN ISS Space Greenhouse in Antarctica During the 2018 Experiment Phase
Source: Front Plant Sci. 2020 May 26;11:656. doi: 10.3389/fpls.2020.00656 (PMC7264257; doi:10.3389/fpls.2020.00656)
Supplement: Supplementary file 1 [file Table_1.DOCX]

Supplementary Material

# Supplementary Figures and Tables

The following table lists all crops grown in the EDEN ISS greenhouse in the 2019 experiment phase. The crop category, commonly used name, scientific name and seed supplier are shown for each cultivar.

| **Crop category** | **Common name** | **Scientific name** | **Seed supplier** |
| --- | --- | --- | --- |
| Lettuce | Batavia | *Lactuca sativa* | Rijk Zwaan |
| Lettuce | Expertise | *Lactuca sativa* | Rijk Zwaan |
| Lettuce | Outredgeous | *Lactuca sativa* | Johnny’s Selected Seeds |
| Lettuce | Waldmann’s Green | *Lactuca sativa* | Johnny’s Selected Seeds |
| Leafy Greens | Red Giant | *Brassica juncea* | Rühlemann’s Kräuter & Duftpflanzen |
| Leafy Greens | Frizzy Lizzy | *Brassica juncea (L.) Czern.* | Hild Samen GmbH |
| Leafy Greens | Swiss Chard | *Beta vulgaris* | Johnny’s Selected Seeds |
| Leafy Greens | Arugula | *Eruca vesicaria* subsp. *sativa* | Franchi Sementi |
| Leafy Greens | Mizuna | *Brassica rapa* | Rühlemann’s Kräuter & Duftpflanzen |
| Herbs | Basil Dolly | *Ocimum basilicum* | Johnny’s Selected Seeds |
| Herbs | Parsley | *Petroselinum crispum* | Rijk Zwaan |
| Herbs | Chives | *Allium schoenoprasum* | Johnny’s Selected Seeds |
| Fruit Crops | Tomato F1 3496B | *Lycopersicon esculentum* | Vreugdenhil |
| Fruit Crops | Tomato F1 1202 | *Lycopersicon esculentum* | Vreugdenhil |
| Fruit Crops | Cucumber Picowell | *Cucumis sativus* | Rijk Zwaan |
| Tuber Crops | Radish Raxe | *Raphanus sativus L. var. sativus* | Hild Samen GmbH |
| Tuber Crops | Radish Lennox | *Raphanus sativus L. var. sativus* | Hild Samen GmbH |
| Tuber Crops | Kohlrabi | *Brassica oleracea var. gongylodes L.* | Gärtner Pötschke |
| Misc. Crops | Cilantro | *Coriandrum sativum* | Hild Samen GmbH |
| Misc. Crops | Mint | *Mentha piperita* | Gärtner Pötschke |
| Misc. Crops | Lemon Balm | *Melissa officinalis* | Rühlemann’s Kräuter & Duftpflanzen |
| Misc. Crops | Celery | *Apium graveolens* | Gärtner Pötschke |
| Misc. Crops | Pepper 1601-M | *Capsicum annuum* | Vreugdenhil |
| Misc. Crops | Pepper Cupid | *Capsicum annuum* | Johnny’s Selected Seeds |
| Misc. Crops | Tomato Bogus Fruchta | *Lycopersicon esculentum* | bingenheimer saatgut |
| Misc. Crops | Tomato Harzfeuer | *Lycopersicon esculentum* | Sperli |
| Misc. Crops | Strawberry | Unknown. | Dürr Samen |
